# Fusion of GNSS and Speedometer Based on VMD and Its Application in Bridge Deformation Monitoring

**DOI:** 10.3390/s20030694

**Published:** 2020-01-27

**Authors:** Ruicheng Zhang, Chengfa Gao, Shuguo Pan, Rui Shang

**Affiliations:** 1 School of Transportation, Southeast University, Nanjing 211189, China; zrc_1996@seu.edu.cn (R.Z.); shangrui1994@foxmail.com (R.S.); 2 School of Instrument Science and Engineering, Southeast University, Nanjing 210096, China

**Keywords:** GNSS, speedometer, VMD, dynamic displacement, bridge deformation monitoring

## Abstract

Real-time dynamic displacement and spectral response on the midspan of Jiangyin Bridge were calculated using Global Navigation Satellite System (GNSS) and a speedometer for the purpose of understanding the dynamic behavior and the temporal evolution of the bridge structure. Considering that the GNSS measurement noise is large and the velocity/acceleration sensors cannot measure the low-frequency displacement, the Variational Mode Decomposition (VMD) algorithm was used to extract the low-frequency displacement of GNSS. Then, the low-frequency displacement extracted from the GNSS time series and the high-frequency vibration calculated by speedometer were combined in this paper in order to obtain the high precision three-dimensional dynamic displacement of the bridge in real time. Simulation experiment and measured data show that the VMD algorithm could effectively resist the modal aliasing caused by noise and discontinuous signals compared with the commonly used Empirical Mode Decomposition (EMD) algorithm, which is guaranteed to get high-precision fusion data. Finally, the fused displacement results can identify high-frequency vibrations and low-frequency displacements of a mm level, which can be used to calculate the spectral characteristics of the bridge and provide reference to evaluate the dynamic and static loads, and the health status of the bridge in the full frequency domain and the full time domain.

## 1. Introduction

Large-scale bridges are the transport lifeline of a country or region, and they cause significant personnel and economic losses when damage occurs. However, various types of damage and functional degradation are inevitable as the service life of the bridge and the traffic load increase [1]. In order to understand the health of large bridges in time, and to issue early warning information before an irreversible accident occurs, it is necessary to adopt a scientific method to conduct real-time safety monitoring of the bridge during the operation period.

There are many traditional types of equipment for bridge deformation monitoring including speedometer/accelerometer, deflection meter, and inclination sensors, etc. However, the above-mentioned devices also have great shortcomings, such as large workload, cumbersome testing, being restricted by the observation environment, and inability to continuously observe for a long time. This also causes the low-frequency displacement of the bridge, which then cannot be effectively monitored [2]. On the contrary, GNSS (Global Navigation Satellite System) technology can overcome all these shortcomings [3,4,5], and it has been widely used in bridge deformation monitoring since 1996 [6]. Nevertheless, GNSS has a problem of severe noise, and it is difficult to meet the millimeter-level accuracy requirements of deformation monitoring by simply using GNSS technology. Therefore, many scholars try to combine GNSS with other sensors, such as speedometer/accelerometer [7,8,9]. Yu et al. [10] adopted the improved adaptive multi-mode filter algorithm (MAF) to process the data of the mid-span bridge, and compared it with the accelerometer results to achieve sub-millimeter-level monitoring accuracy. Koo et al. [11] used a low-cost RTK (real-time kinematic) and accelerometer fusion device to test with a vibrating table, achieving an accuracy of 2 mm. 

In order to further reduce GNSS time series noise and extract trend items, filtering algorithms were used such as empirical mode decomposition (EMD), a signal processing method that can be used for nonlinear time series processing [12]. In Shen et al. [13], EMD is used to decompose the GNSS measurement sequence and acceleration measurement sequence into a series of IMF (Intrinsic Mode Function) components and final residuals, respectively. In Chan et al. [14], to verify the accuracy and effectiveness of the combined algorithms using AF (Adaptive Filter) and EMD, a series of experiments based on a motion simulation table were performed. Ke et al. [15] studied the EMD for decomposing displacement for further processing. In Chao et al. [16], a combined model of EMD and wavelet is applied to extract the systematic errors from the residual series of double difference observation. However, because EMD has problems such as modal aliasing and over-envelope, it is difficult for EMD to effectively decompose useful signals when the original signal has high-frequency vibration signals locally.

In this paper, an adaptive time-frequency decomposition technique, variational mode decomposition (VMD), is implemented for extracting low-frequency trend items from GNSS time series, which is a nonrecursive method proposed by K. Dragomiretskiy [17]. It can adaptively extract the intrinsic modes from any nonlinear and nonstationary signals concurrently. Due to the frequency domain non-recursive solution method, the decomposition accuracy is high and the modal aliasing problem can be well avoided. It has been widely used for mechanical fault diagnosis [18,19], which is the estimation and mitigation of ionospheric scintillation effects on GNSS signal [20]. However, there is almost no research on GNSS displacement time series and bridge deformation monitoring. 

To increase the measurement accuracy of the bridge dynamic displacement, the low-frequency movements extracted from GNSS by the VMD and the vibration information calculated by speedometer were combined together. The simulation data and the measured data of Jiangyin Bridge all verified the feasibility and reliability of the algorithm. In this paper, Section 2 and Section 3 introduces the VMD and fusion algorithm for GNSS and speedometer data. Section 4 discusses the experiment results and engineering applications, and Section 5 presents the conclusions.

## 2. The Principle of the VMD Algorithm

### 2.1. The Derivation of the VMD Algorithm

The VMD begins with a simple denoising problem: separate the original signal f(t) from signal $f_{0}(t)$, which is affected by additive zero-mean Gaussian noise:

(1) $$f_{0}=f+\eta .$$

Tikhonov regularization is adopted to solve this typical ill-posed inverse problem [17], meanwhile, signals are transformed to the complex field by Fourier transform to solve the differential conveniently.

(2) $$\underset{\hat{f}(\omega )}{\min}\left\{{\left\|\hat{f}(\omega )-{\hat{f}}_{0}(\omega )\right\|}_{2}^{2}+\alpha {\left\|j\omega \hat{f}(\omega )\right\|}_{2}^{2}\right\}.$$

The extreme value can be obtained by expanding the above formula into functional and deriving the partial derivative of $\hat{f}$.

(3) $$J[\hat{f}]=\int \left\{{\left(\hat{f}(\omega )-{\hat{f}}_{0}(\omega )\right)}^{2}+\alpha \omega^{2}{\left(\hat{f}(\omega )\right)}^{2}\right\}d\omega$$

(4) $$\frac{\delta J[\hat{f}]}{\delta \hat{f}}=2\left(\hat{f}-{\hat{f}}_{0}\right)+2\alpha \omega^{2}\hat{f}=0\Rightarrow \hat{f}=\frac{{\hat{f}}_{0}}{1+\alpha \omega^{2}}$$

where $\hat{f}(\omega )=F\{f(\cdot )\}(\omega )=\frac{1}{\sqrt{2\pi }}\int f(t)e^{-j\omega t}dt$, with $j^{2}=-1$ (j is imaginary unit), is the Fourier transform of the signal f(t). $J[\hat{f}]$ is the result of functional expansion. It can be seen that the obtained f is equivalent to filtering the observation signal in the frequency band of $f_{0}$, and filtering out the high-frequency part and obtaining a low-pass narrow-band selection. 

### 2.2. The Process of the VMD Algorithm

The whole framework of the VMD is a variational problem, finding the maximum or minimum of function, which mainly includes the construction of variational problem and its solution process. It is assumed that the input signal f is decomposed into K modal functions $u_{k}(t)$, each of which is a Band-Limited Intrinsic Mode Function (BLIMF) with a central frequency and a finite bandwidth. In order to achieve the goal of estimating the frequency bandwidth of IMF components, the following framework is defined [17]:

1. For each mode function $u_{k}$, the marginal spectrum is obtained by Hilbert transform;
2. Using exponential correction, the frequency spectrum of modal function is moved to the center frequency of each estimation;
3. The signal is demodulated by Gaussian smoothing (the square root of $L^{2}$ norm gradient) to obtain the bandwidth of each modal function.

In order to minimize the sum of the bandwidth estimates of each mode, the signal f is equal with the sum of all modes, and the constrained variational problem is established as follows:

(5) $$\begin{array}{c} \underset{\left\{u_{k}\},\{\omega_{k}\right\}}{\min}\left\{\sum_{k=1}^{K}{\left\|\partial_{t}\left[\left(\delta \left(t\right)+\frac{j}{\pi t}\right)*u_{k}(t)\right]e^{-j\omega_{k}t}\right\|}_{2}^{2}\right\}\\ s.t.\sum_{k=1}^{K}u_{k}(t)=f(t) \end{array}$$

where $\left\{u_{k}\right\}=\left\{u_{1},\cdots u_{k}\right\},\left\{\omega_{k}\right\}=\left\{\omega_{1},\cdots \omega_{k}\right\}$ are the set of all mode functions and their center frequencies, respectively; $\partial_{t}$ is the partial derivative of time t for function; δ(t) is the pulse function; and * represents convolution.

For rendering the problem unconstrained, both a quadratic penalty term, α, and Lagrangian multipliers, λ, are applied. The extended Lagrangian expression is as follows:

(6) $$\begin{array}{c} L\left(\left\{u_{k}\right\},\left\{\omega_{k}\right\},\lambda \right)=\alpha {\sum_{k=1}^{K}\left\|\partial_{t}\left[\left(\delta \left(t\right)+\frac{j}{\pi t}\right)*u_{k}(t)\right]e^{-j\omega_{k}t}\right\|}_{2}^{2}\\ +{\left\|f\left(t\right)-\sum_{k=1}^{K}u_{k}(t)\right\|}_{2}^{2}+\langle \lambda \left(t\right),f\left(t\right)-\sum_{k=1}^{K}u_{k}(t)\rangle \end{array}$$

Then, the alternate direction method of multipliers (ADMM) has been used to solve Equation (6) from the saddle point of the above-augmented Lagrange function, which can find the central frequencies and IMFs centered around those frequencies simultaneously, and among which $u_{k}^{n+1}$ can be obtained from the following equation.

(7) $$\begin{array}{cc} u_{k}^{n+1} & =\underset{u_{k}\in X}{\arg\min}\{\alpha {\left\|\partial_{t}\left[\left(\delta \left(t\right)+\frac{j}{\pi t}\right)*u_{k}(t)\right]e^{-j\omega_{k}t}\right\|}_{2}^{2}\\ & +{\left\|f\left(t\right)-\sum_{i}u_{i}(t)+\frac{\lambda \left(t\right)}{2}\right\|}_{2}^{2}\} \end{array}$$

Equation (7) is transformed to frequency domain by using the Parseval/Plancherel Fourier equidistant transformation, and the solution of the quadratic optimization problem is obtained:

(8) $${\hat{u}}_{k}^{n+1}\left(\omega \right)=\frac{\hat{f}\left(\omega \right)-\sum_{i\neq k}{\hat{u}}_{i}\left(\omega \right)+\frac{{\hat{\lambda }}^{n}\left(\omega \right)}{2}}{1+2\alpha {\left(\omega -\omega_{k}^{n}\right)}^{2}}$$

(9) $${\hat{\lambda }}^{n+1}\left(\omega \right)={\hat{\lambda }}^{n}\left(\omega \right)+\tau \left(\hat{f}\left(\omega \right)-\sum_{k=1}^{K}{\hat{u}}_{k}^{n+1}(\omega )\right).$$

Similarly, the minimum value of $\omega_{k}^{n+1}$ can be obtained as follows:

(10) $$\omega_{k}^{n+1}=\frac{\int_{o}^{\infty }\omega {\left|{\hat{u}}_{k}^{n+1}\left(\omega \right)\right|}^{2}d\omega }{\int_{o}^{\infty }{\left|{\hat{u}}_{k}^{n+1}\left(\omega \right)\right|}^{2}d\omega }$$

where ${\hat{u}}_{k}^{n+1}\left(\omega \right)$ is the winner filter of current surplus $\hat{f}\left(\omega \right)-\sum_{i\neq k}{\hat{u}}_{i}\left(\omega \right)$; $\omega_{k}^{n+1}$ is the center of gravity of the modal power spectrum. By inverse Fourier transform of ${\hat{u}}_{k}^{}\left(\omega \right)$, the real part of the result is the time domain modal component $u_{k}\left(\omega \right)$.

### 2.3. The VMD Algorithm to Decompose the GNSS Time Series (Algorithm 1)

**Algorithm 1:** The VMD algorithm to decompose the GNSS time series**Initialize**$\left\{{\hat{u}}_{k}^{1}\right\},\left\{{\hat{\omega }}_{k}^{1}\right\},{\hat{\lambda }}^{1},n\leftarrow 0$, where n is the iteration number.**repeat** the entire cycle, n←n+1.  **For**
k=1:K
**do**    Update ${\hat{u}}_{k}$ for all w≥0, by Equation (8);    Update $\omega_{k}$, by Equation (10);  **end for**  Do dual ascent for all w≥0, by Equation (9);**until** Iterative constraints satisfied: ${\sum_{k}{\left\|{\hat{u}}_{k}^{n+1}-{\hat{u}}_{k}^{n}\right\|}_{2}^{2}/\left\|{\hat{u}}_{k}^{n}\right\|}_{2}^{2}<\varepsilon$

## 3. Integrating the Displacement of GNSS and Speedometer

### 3.1. Decompose the GNSS Displacement Time Series by the VMD

Based on the characteristics of bridge vibration, the GNSS displacement time series can be divided into three parts based on characteristics (frequency and amplitude, etc.): low-frequency trend term (denoted by $G_{l}$), vibration signal (denoted by $G_{m}$), and high-frequency noise (denoted by $G_{h}$).

- **Low-frequency trend:** this component mainly consists of the low-frequency displacement of the bridge and the multi-path effect of GNSS (caused by the span wires and the vehicles); after the multi-path is weakened by the appropriate algorithm, this component can reflect the low-frequency displacement of the bridge accurately.
- **Vibration signal:** this component reflects the vibration of the bridge structure, and the frequency is generally between 0.5 and 5 Hz; the amplitude is several centimeters or millimeters, which can be used to judge the health of the bridge.
- **High-frequency noise:** this component has the highest frequency among all the three components, which is caused by the receiver and GNSS technology itself, and cannot be eliminated directly during the positioning solution, which is generally up to mm level.

Based on the above analysis, the parameter *K* in the VMD algorithm is set to 3. In addition, due to the poor accuracy of the VMD algorithm for high-frequency signal extraction, the vibration signal $G_{m}$ may not meet the accuracy requirements of bridge health monitoring. Therefore, in this paper, the $G_{m}$ is replaced by the vibration information obtained by the speedometer integration.

### 3.2. Data Fusion of GNSS Low-Frequency Trend and Speedometer Displacement

Data fusion is not simply adding the GNSS low-frequency trend term and the displacement obtained by speedometer integration, but also considering the unity of time, coordinate system, and sampling rate. Only in this way can the fusion result be meaningful.

- **The unity of sampling rate**

The sampling frequency of a GNSS receiver is generally dozens of Hz, and the sampling rate of a speedometer is hundreds or even thousands of Hz. Therefore, we can make the sampling rate consistent by interpolation of the GNSS data or downsampling the speedometer data.

- **The unity of coordinate system**

**Figure 1:**
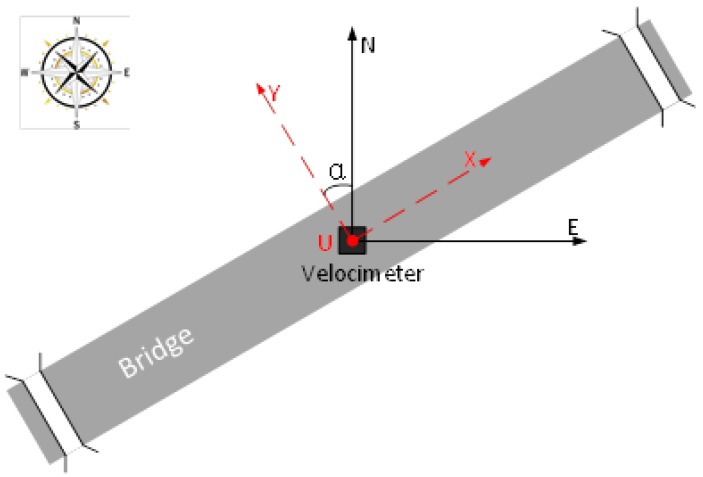
Equipment installation diagram on the bridge.

GNSS and speedometer use the geocentric coordinate system and carrier coordinate system, respectively, which need to be unified by certain coordinate conversion methods. Due to the small volume of the speedometer, direct measurement of its orientation will cause a large error. Therefore, when installing the speedometer on the bridge, two of its axes are respectively pointed to the axial and transverse direction of the bridge, and the other axis is pointed to the direction of the zenith (U direction) through the leveling device (Figure 1). In this way, the two coordinate systems can be consistent through plane coordinate transformation only, as shown in Equation (11):

(11) $$\left[\begin{array}{c} X\\ Y \end{array}\right]=\left[\begin{array}{cc} \cos\left(\alpha \right) & -\sin\left(\alpha \right)\\ \sin\left(\alpha \right) & \cos\left(\alpha \right) \end{array}\right]\cdot \left[\begin{array}{c} E\\ N \end{array}\right]$$

- **The unity of time**

The output data of the speedometer can be time tagged by the second pulse of GNSS, which can be realized at the hardware, so that the time system of the two sensors can be unified. After that, the accuracy of time synchronization needs to be evaluated. In this paper, the accuracy of time synchronization is evaluated by calculating the correlation coefficient between the GNSS vibration signal and the displacement of the speedometer. Based on the consistency of the sampling rate and the coordinate system, the specific Algorithm 2 is as follows.


**Figure 2:**
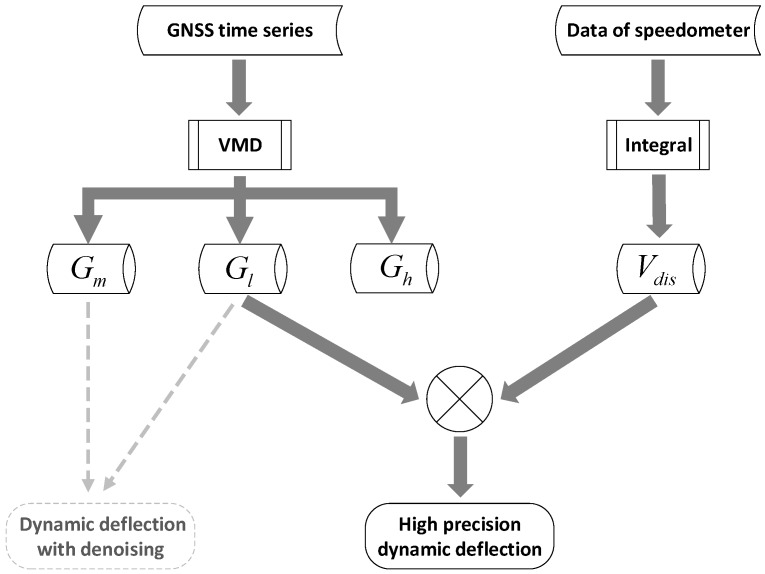
The algorithm flow of data fusion.

**Algorithm 2: Evaluate the Accuracy of Time Synchronization**
Get GNSS low-frequency trend $G_{l}$ by the VMD, Get speedometer displacement $V_{dis}$ by integral(Time period of $G_{l}$ is included in $V_{dis}$);**Initialize**: $N_{G}\leftarrow size(G_{l})$, $N_{V}\leftarrow size(V_{dis})$, corre_max←0, corre_max_n←0;**For**$n=1:(N_{V}-N_{G})$ do  $temp\leftarrow corr(G_{l},V_{dis}(n:n+N_{G}-1))$  **If**
temp>corre_max    corre_max←temp,corre_max_n←n;  **End if** **End for****Return**corre_max, corre_max_n;

After all the preparations are completed, GNSS and speedometer results can be integrated, the specific process is shown in Figure 2.

## 4. Results

In order to verify the feasibility and reliability of the VMD algorithm in the GNSS time series decomposition, this paper compares and analyzes the effects of the VMD and the EMD algorithms in simulation data, test data, and actual bridge vibration data. In addition, according to the algorithm in Section 2 and Section 3, the GNSS and speedometer results in the test data and bridge vibration data are combined, and the time-frequency characteristics and accuracy of the dynamic deflection before and after fusion are compared.

### 4.1. The Results of Simulation Data

The modal aliasing problem is a common problem in EMD. The two adjacent modal functions interfere with each other, which makes waveform aliasing difficult to distinguish, and this is the main reason why EMD is not ideal when dealing with actual data [21]. Huang et al. [22] believe that intermittent phenomena are the main cause of modal aliasing, but abnormal signals, such as noise signals and discontinuous signals, are usually caused by intermittent phenomena. Therefore, in this paper, the simulation analysis of the VMD and the EMD is carried out on the simulation signals composed of noise signals and discontinuous signals, respectively, to verify the advantages of the VMD method.

#### 4.1.1. Signal Without Noise

First of all, the decomposition of the EMD and the VMD for pure signals without noise are tested. The analog signal S used for decomposition consists of three different frequency, amplitude sine, or cosine functions, and their expressions and waveforms are as follows:

(12) $$\left\{ \begin{array}{c} samplingrate=20Hz,time=20s\\ f_{1}=0.1Hz,f_{2}=2Hz,f_{3}=5Hz\\ S_{1}=\cos\left(2*\pi *f_{1}*t\right),S_{2}=0.5*\sin\left(2*\pi *f_{2}*t\right)\\ S_{3}=0.2*\sin\left(2*\pi *f_{3}*t+\pi /5\right)\\ S=S_{1}+S_{2}+S_{3} \end{array}\right.$$

**Figure 3:**
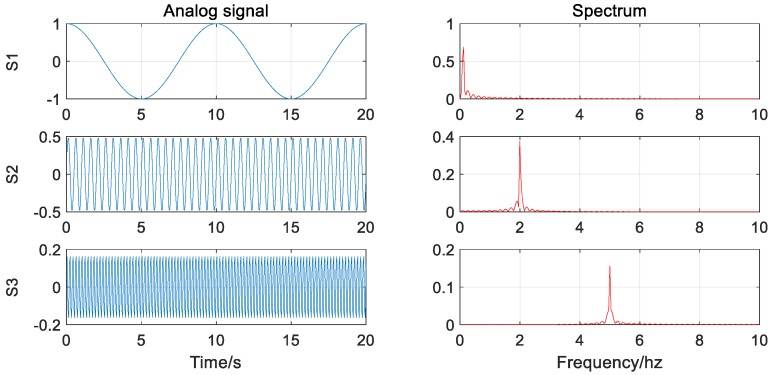
Signal waveform and corresponding spectrogram (without noise).

**Figure 4:**
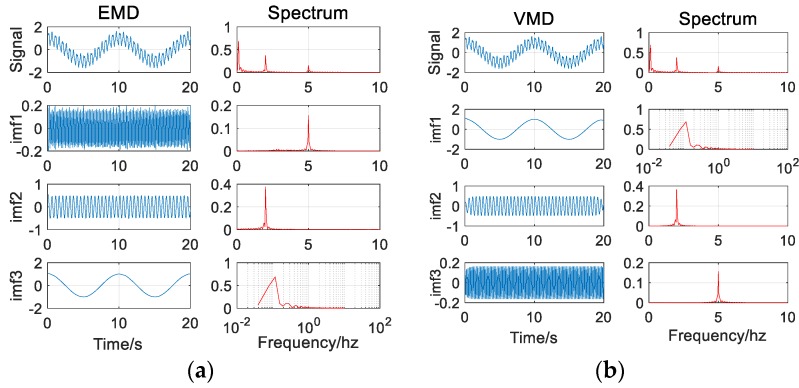
(**a**) The EMD (Empirical Mode Decomposition) decomposition results of the signal (without noise); (**b**) The VMD (Variational Mode Decomposition) decomposition results of the signal (without noise).

It can be seen from the decomposition results that in the absence of noise (as in Figure 3), both methods can effectively and accurately decompose the signal components without modal aliasing. In addition, for high-frequency signal components, the VMD decomposition results are more stable than the EMD decomposition results (as in Figure 4).

#### 4.1.2. Signal with Noise

**Figure 5:**
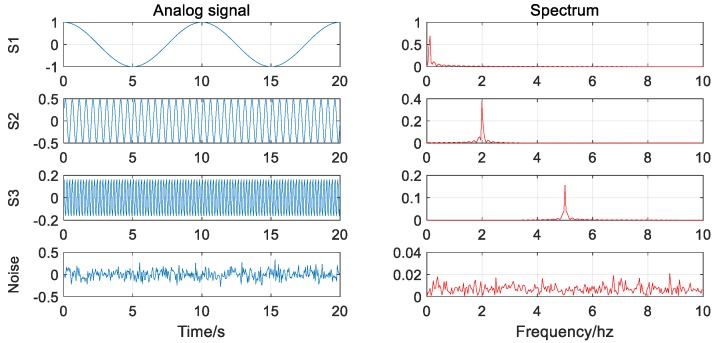
Signal waveform and corresponding spectrogram (with noise).

Then, in order to verify whether the EMD and the VMD algorithms will exhibit the above-mentioned modal aliasing, noise was added based on Equation (13). The signal with noise and its spectrum are shown in Figure 5.

(13) $$S=S_{1}+S_{2}+S_{3}+0.1*randn(size(S_{1})).$$

**Figure 6:**
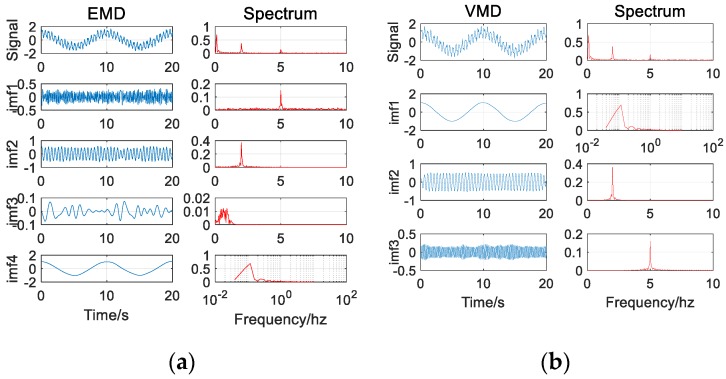
(**a**) The EMD decomposition results of the signal (with noise); (**b**) The VMD decomposition results of the signal (with noise).

It can be seen from the decomposition results (Figure 6) that the addition of noise causes the EMD algorithm to be over-decomposed compared to the case of no noise, and there is an IMF3 component (about 1 Hz) that is not present in the actual signal. The VMD algorithm is almost immune to noise and completely decomposes the individual signal components.

#### 4.1.3. Discontinuous Signal

**Figure 7:**
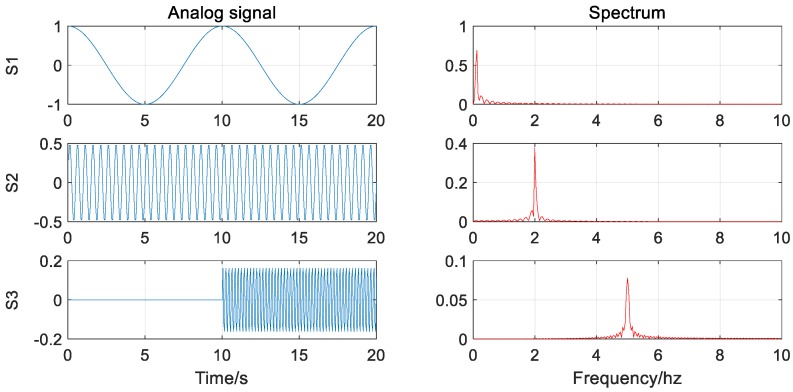
Signal waveform and corresponding spectrogram (with discontinuous signal).

Finally, the signal component $S_{3}$ is changed to a discontinuous signal, which is also common in the actual vibration signal of the bridge, to verify whether the VMD algorithm can be applied to the actual environment. The discontinuous signal and its spectrum are shown in Figure 7.

(14) $$S_{3}=\{\begin{array}{cc} 0 & t\leq 200\\ 0.2*\sin\left(2*\pi *f_{3}*t+\pi /5\right) & t>200 \end{array}.$$

**Figure 8:**
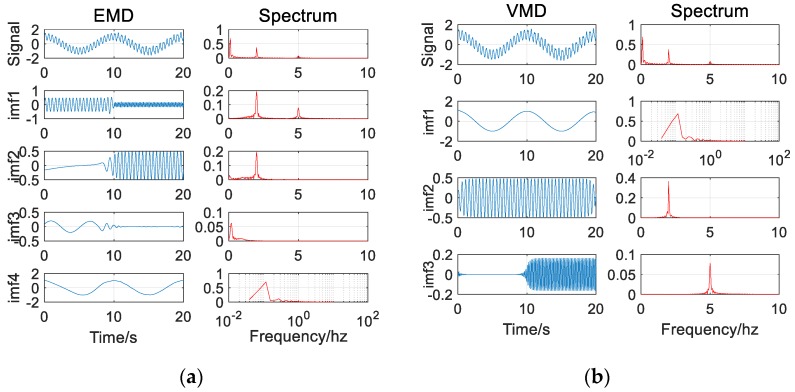
(**a**) The EMD decomposition results of the signal (with discontinuous signal); (**b**) The VMD decomposition results of the signal (with discontinuous signal).

Similar to the addition of noise, the introduction of discontinuous signals also causes severe modal aliasing problems in the EMD decomposition results. Although there are certain short-point problems in the VMD algorithm, the decomposition accuracy is still within the acceptable range, especially for the low-frequency components that we pay more attention to (as in Figure 8).

**Figure 9:**
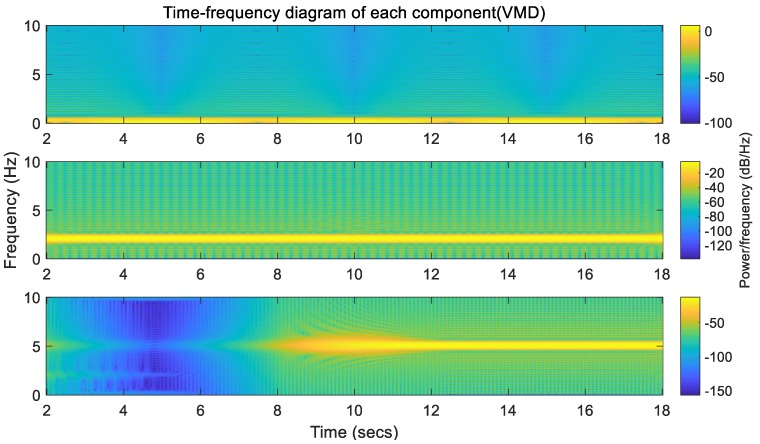
Time-frequency diagram of each component (VMD).

Time-frequency analysis is used to view the changes of signals in both the time domain and the frequency domain, and is performed on the three signal components decomposed by the VMD. As shown in Figure 9, the horizontal axis is time, the vertical axis is frequency, and the yellow highlighted part is the signal. It can be seen from the figure that the frequency and distribution in the time domain of three signal components are consistent with the experimental design. In addition, the endpoint effect of the VMD algorithm can also be seen from the figure clearly (the signal component should appear at 10th second, but it appears at 8th second).

### 4.2. The Results of Measured Data

**Figure 10:**
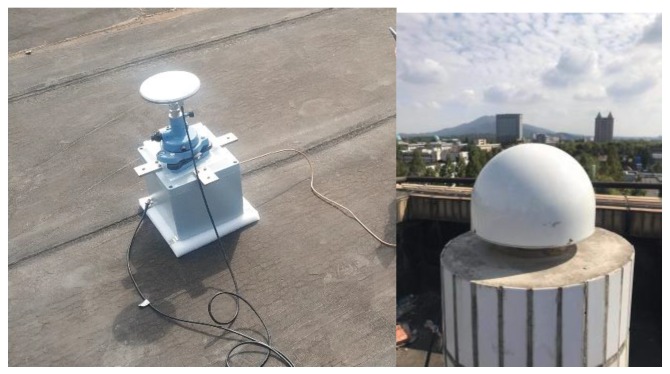
Experimental environment and equipment placement.

In order to compare the feasibility and reliability of the EMD and the VMD for GNSS time series decomposition, we conducted the following experiments on the wider roof (reducing the effect of multipath effect). First, we placed the speed sensor on the ground with the X-axis facing north (which can omit the coordinate conversion step). Then, we placed the antenna with the base on the speed sensor, fixed it and leveled it. Finally, we moved the device along the X-axis according to a certain amplitude and frequency to simulate the vibration signal. The experimental environment and equipment placement are shown in Figure 10. Specific equipment and data information are shown in Table 1.

**Figure 11:**
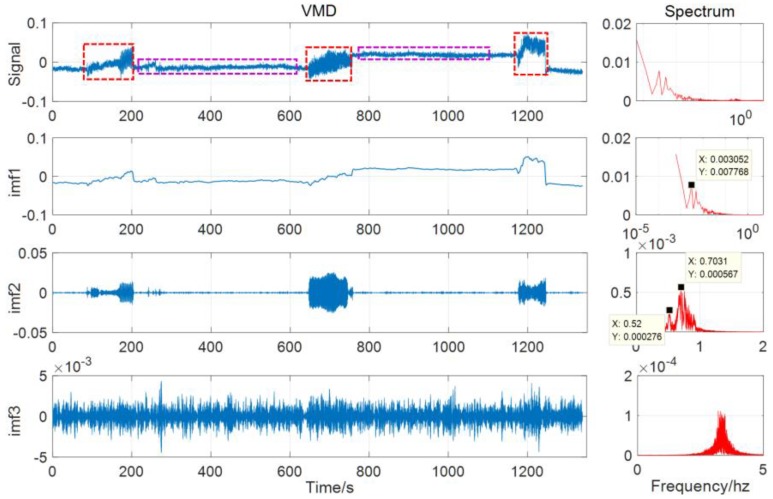
The VMD result of GNSS (Global Navigation Satellite System) (the vertical axis represents the displacement/m).

Since vibration and displacement are mainly generated in the X direction (N direction of GNSS), the result analysis is performed only for the X direction below. First of all, the GNSS time series is obtained by the RTK algorithm [23,24,25]. It can be seen from the GNSS time series (as in Figure 11) that the vibration mainly occurs in three time periods (red dotted frame), and the device has a significant permanent displacement before and after the vibration (purple dotted frame), which is the low-frequency trend item mentioned earlier.

**Figure 12:**
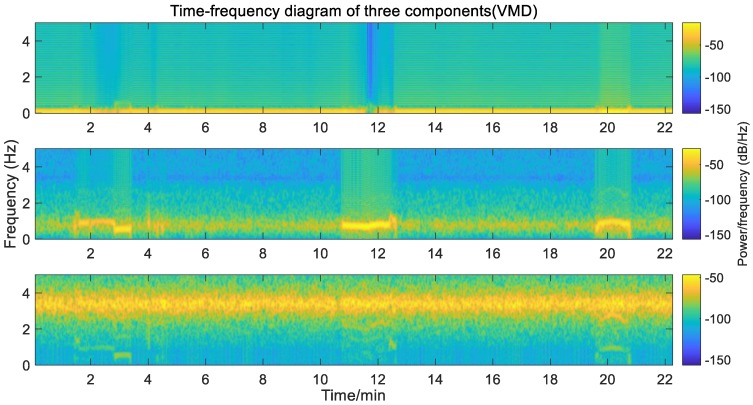
Time-frequency diagram of three components (GNSS displacement time series).

Then, the GNSS time series is decomposed, and according to the analysis in Section 3.1, three signal components can be obtained. They are low-frequency displacement (IMF1), vibration signal (IMF2, around 0.5 Hz and 0.7 Hz), and high-frequency noise (IMF3). Combined with the time-frequency diagram (as in Figure 12), it can be seen that the VMD algorithm clearly decomposes the components of different frequencies and better restores the actual vibration.

**Figure 13:**
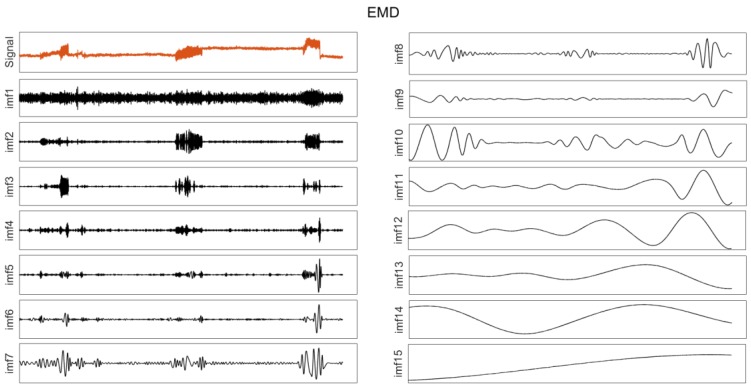
The EMD result of GNSS.

At the same time, the EMD algorithm was used to decompose the same group of data, and the results are shown in Figure 13. The orange line represents the original signal, and the black lines represent the decomposed components, from IMF1 to IMF15. The horizontal axis represents the time (Time/s), and the vertical axis represents the displacement (Displacement/m). It is obvious that the modal aliasing phenomenon occurs in the results of the EMD algorithm; therefore, the actual vibration cannot be recognized from the components. 

Furthermore, the correlation between the IMF2 component in the VMD decomposition result and the displacement obtained by the speedometer integration is analyzed to verify the accuracy of time synchronization and to prove the correctness of the VMD decomposition result. 

**Figure 14:**
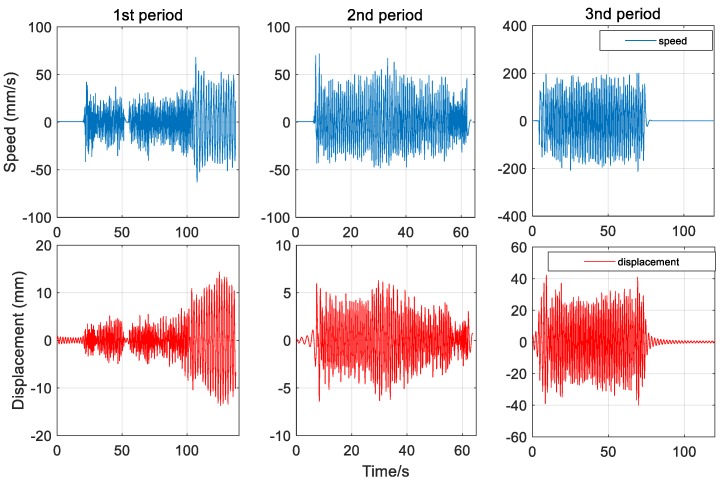
Integration results of the speedometer for three periods.

**Figure 15:**
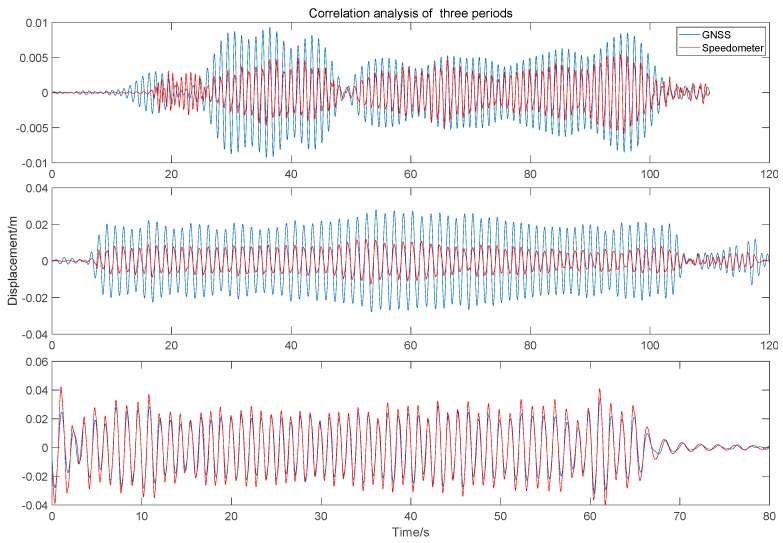
Correlation analysis of integral displacement of the speedometer and IMF2 component in three periods.

Figure 14 shows the speed data of three vibration period obtained by speedometer, and the corresponding displacement calculated by integral. The results show that their correlation coefficients with IMF2 of GNSS are 96.5%, 96.4%, and 98.0%, respectively. The comparison diagram of local amplification of vibration signals in three periods is shown in Figure 15. 

**Figure 16:**
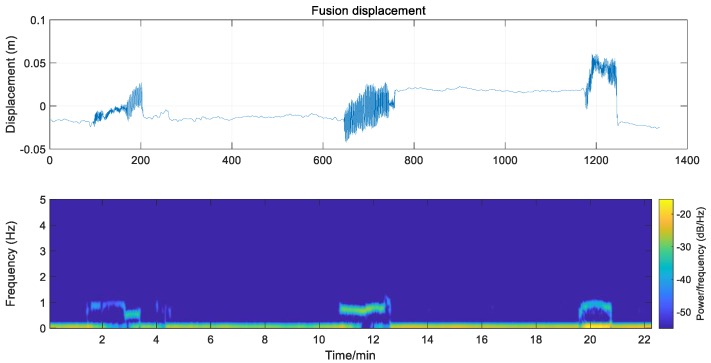
The fusion displacement of GNSS and speedometer.

Finally, by adding the IMF1 component of GNSS after the unifying the sampling rate, time, and coordinate system with the integral displacement of the speedometer, the fusion displacement results can be obtained, as shown in Figure 16. 

### 4.3. Algorithm Applied in Jiangyin Bridge

**Figure 17:**
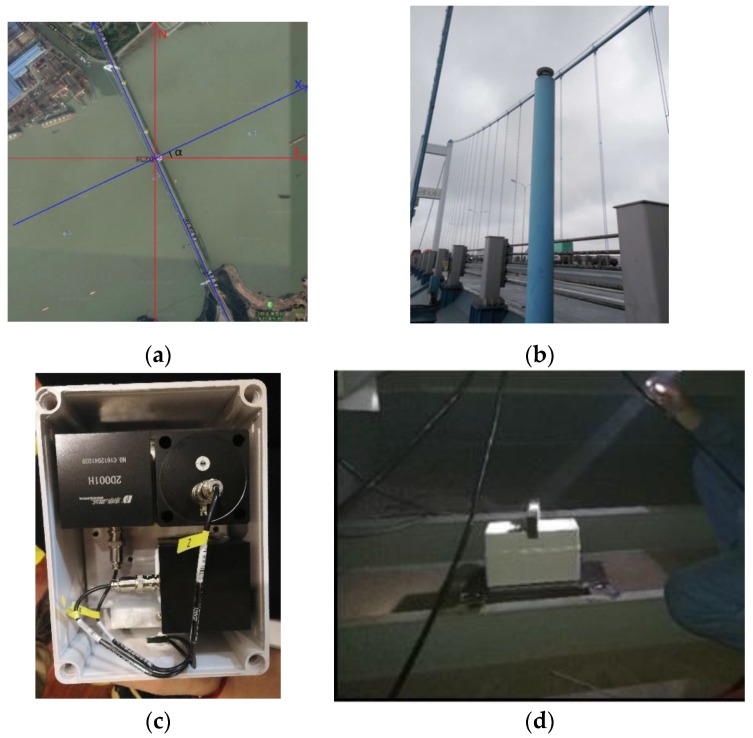
Orientation of Jiangyin Bridge and equipment installation. (**a**) Satellite image of Jiangyin Bridge and coordinate system relationship; (**b**) GNSS antenna position and observation pier; (**c**) the internal of Speedometer; (**d**) speedometer installation.

In order to further verify the performance of the proposed algorithm in the actual environment of the bridge, the fusion algorithm and self-developed equipment were applied to the monitoring of Jiangyin Bridge. First of all, the angle between the local Cartesian coordinate system (NE) and the carrier coordinate system (XY) is obtained by combining the measured point coordinates and Google map (α≈25°). The GNSS equipment uses the original antenna on the bridge, which is mounted on the observation pier (approx. 2.5 *m*) to reduce the multipath effect of passing vehicles and surrounding structures. However, this also makes the GNSS time series contain a part of the displacement of the observation pier vibration, making it impossible to accurately measure the displacement of the bridge itself (as in Figure 17).

Furthermore, the speedometer is installed in the bridge box, below the GNSS antenna, to measure the high-frequency vibration information of the bridge accurately. The two plane axes of the speedometer face the axial and lateral directions of the bridge, respectively, and it is important to level the air bubbles to ensure that the third axis is consistent with the zenith direction of the GNSS antenna.

#### GNSS Time Series Analysis and Low-Frequency Displacement Extraction With the VMD

**Figure 18:**
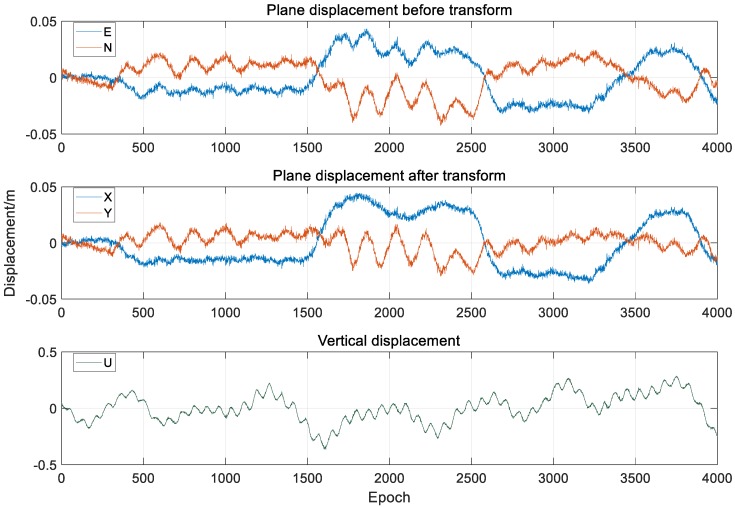
Comparison of displacement before and after coordinate transformation.

After the displacement of the monitoring point obtained by the RTK algorithm [24,25,26], the vibration result is converted into the carrier coordinate system according to the algorithm in Section 3.2. The displacement sequence before and after conversion is shown in Figure 18.

**Figure 19:**
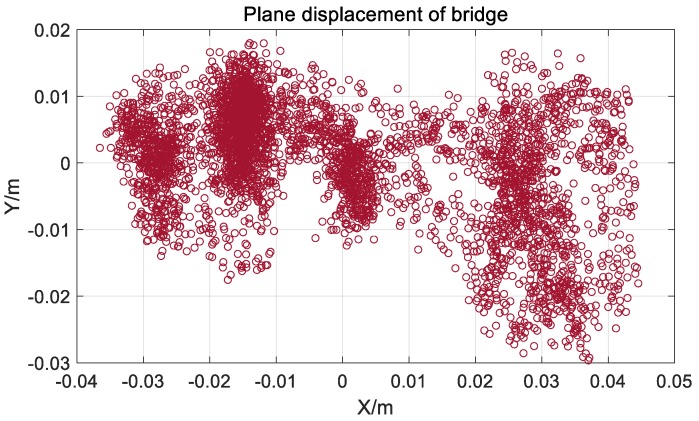
Plane displacement after coordinate transformation of Jiangyin Bridge.

It can be seen from Figure 18 and Figure 19 that the vertical direction has a relatively obvious and large amplitude low-frequency displacement, which is mainly caused by the vehicle load. The lateral displacement is obviously larger than the axial displacement, which is mainly caused by the wind load, and the axial displacement is less, which is in line with the actual bridge vibration characteristics [26].

**Figure 20:**
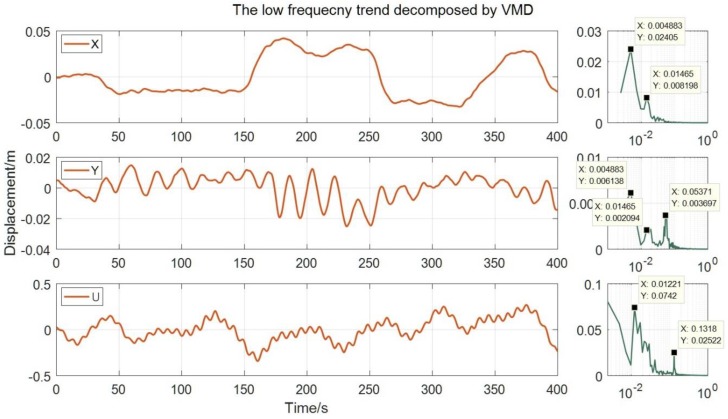
The low-frequency trend decomposed by the VMD and its spectral characteristics.

**Figure 21:**
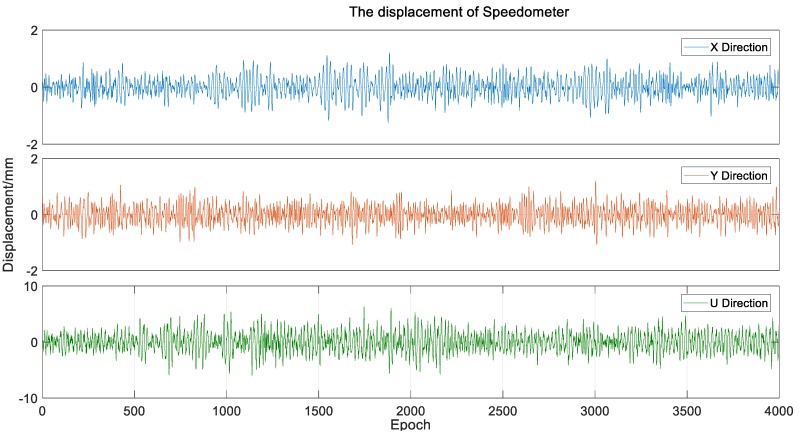
The displacement calculated by the speedometer data.

We utilized spectrum analysis for the low-frequency displacement extracted by the VMD algorithm. As shown in Figure 20 that the peaks appear near 0.0049 Hz and 0.015 Hz both in the axial and lateral directions, which is very difficult to measure the displacement at such a low frequency by a speedometer. The high-frequency vibration calculated by speedometer data is shown in Figure 21, and because Jiangyin Bridge is a long-span suspension bridge, the amplitude of the high-frequency vibration is very small, even less than 1 cm.

**Figure 22:**
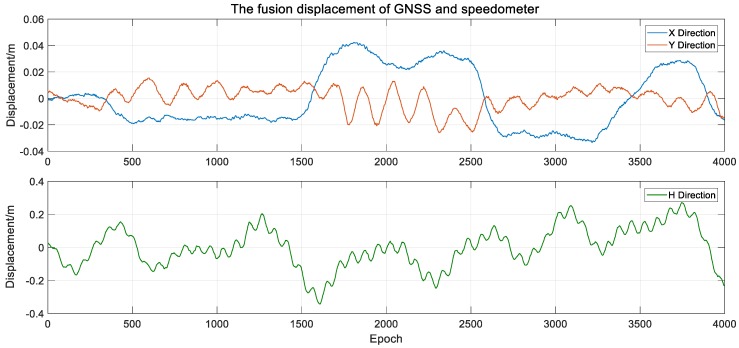
The fusion displacement of GNSS and speedometer.

Using the algorithm in Section 3.2, after data preprocessing, the low-frequency displacement of GNSS extracted by the VMD algorithm and the high-frequency vibration signal obtained by speedometer are superposed, and the fusion three-dimensional dynamic displacement of the monitoring points is obtained. As shown in Figure 22, the noise of the fusion displacement is greatly weakened, and it can effectively reflect the high-frequency vibration and low-frequency displacement of the bridge at the same time, so as to realize the bridge deformation monitoring in full frequency domain and full time domain.

## 5. Discussion

Real-time dynamic displacement and the spectral response of the midspan of Jiangyin Bridge were calculated using the Global Navigation Satellite System (GNSS) and a speedometer. The VMD algorithm was utilized to extract the low-frequency displacement of the GNSS time series. On the basis of the unity of the sampling rate, time system, and coordinate system between GNSS and the speedometer, the displacement, including low-frequency displacement and high-frequency vibration information of bridges, was obtained with high precision. The final displacement can be used to calculate the spectral characteristics of the bridge and to provide reference in order to evaluate the dynamic and static loads, and the health status of the bridge. Some conclusions were reached in this paper:

1) The VMD algorithm used in this paper can effectively resist the modal aliasing phenomenon in the decomposition process caused by noise and discontinuous signals compared with EMD.
2) By a time series analysis and spectrum analysis on the decomposed signal, it is found that the VMD algorithm can extract the low-frequency trend term in the GNSS time series with high precision.
3) The data fusion algorithm proposed in this paper can combine the advantages of two sensors, GNSS and speedometer, and obtains high accuracy displacement including the low-frequency displacement and high-frequency vibration information of bridges.

## Figures and Tables

**Table 1:** Device and data information.

Date	Equipment	Sampling Rate	Remark
2019-10-08	Trimble BD990	10 Hz	Using a measuring antenna as Figure shown
	Magnetoelectric vibration sensor (DH610V)	100 Hz	Timing with GNSS pps
